# Evaluation of a Combined MHE-NMPC Approach to Handle Plant-Model Mismatch in a Rotary Tablet Press

**DOI:** 10.3390/pr9091612

**Published:** 2021-09-08

**Authors:** Yan-Shu Huang, M. Ziyan Sheriff, Sunidhi Bachawala, Marcial Gonzalez, Zoltan K. Nagy, Gintaras V. Reklaitis

**Affiliations:** 1 Davidson School of Chemical Engineering, Purdue University, West Lafayette, IN 47907, USA;; 2 School of Mechanical Engineering, Purdue University, West Lafayette, IN 47907, USA;; 3 Ray W. Herrick Laboratories, Purdue University, West Lafayette, IN 47907, USA

**Keywords:** continuous pharmaceutical manufacturing, model predictive control, state estimation, quality-by-control (QbC), glidant effects, plant-model mismatch

## Abstract

The transition from batch to continuous processes in the pharmaceutical industry has been driven by the potential improvement in process controllability, product quality homogeneity, and reduction of material inventory. A quality-by-control (QbC) approach has been implemented in a variety of pharmaceutical product manufacturing modalities to increase product quality through a three-level hierarchical control structure. In the implementation of the QbC approach it is common practice to simplify control algorithms by utilizing linearized models with constant model parameters. Nonlinear model predictive control (NMPC) can effectively deliver control functionality for highly sensitive variations and nonlinear multiple-input-multiple-output (MIMO) systems, which is essential for the highly regulated pharmaceutical manufacturing industry. This work focuses on developing and implementing NMPC in continuous manufacturing of solid dosage forms. To mitigate control degradation caused by plant-model mismatch, careful monitoring and continuous improvement strategies are studied. When moving horizon estimation (MHE) is integrated with NMPC, historical data in the past time window together with real-time data from the sensor network enable state estimation and accurate tracking of the highly sensitive model parameters. The adaptive model used in the NMPC strategy can compensate for process uncertainties, further reducing plant-model mismatch effects. The nonlinear mechanistic model used in both MHE and NMPC can predict the essential but complex powder properties and provide physical interpretation of abnormal events. The adaptive NMPC implementation and its real-time control performance analysis and practical applicability are demonstrated through a series of illustrative examples that highlight the effectiveness of the proposed approach for different scenarios of plant-model mismatch, while also incorporating glidant effects.

## 1. Introduction

Pharmaceutical manufacturing processes have traditionally employed the batch operation mode, in which fixed amounts of raw materials are run through different unit operations to obtain the final drug product. Quality attributes of the final drug product were originally tested at the end of each batch processing step, where quality control essentially followed a quality-by-testing approach (QbT) [1], e.g., mixing uniformity is tested at the conclusion of the blending process. Over the last few years several factors have driven a shift from batch towards continuous pharmaceutical manufacturing. These factors include a reduction in the development cost for new medicines, making it both desirable and feasible to produce smaller annual volumes of targeted solutions for smaller patient populations, as well as improving product quality, decreasing cycle time, and better controlled processing, to name a few popular drivers [2]. An economic analysis provided by Schaber and co-workers [3] highlights that continuous operation is able to provide estimated overall savings that can range from 9 to 40%, depending on the drug loading and process chosen, when compared to traditional batch operation. Additionally, encouraged by the regulatory agencies to modernize pharmaceutical manufacturing processes, academia and industry have invested significant time and resources to study different aspects required to successfully shift from batch to continuous operation mode. These efforts were made possible through various collaborations and consortiums [4–6].

In 2012, Gernaey and co-workers [7] identified the design and implementation of continuous pharmaceutical processes as one of the many issues that remain unresolved. Advanced process understanding is critical to the implementation of continuous pharmaceutical manufacturing applications [8]. To address this requirement, a quality-by-design (QbD) approach was pursued over the last decade [9]. QbD is a multi-step procedure that involves: (i) definition of quality target product profiles (QTPPs) and critical quality attributes (CQAs), (ii) identification of critical material attributes (CMAs) and critical process parameters (CPPs), (iii) linking of the CMAs and CPPs with the CQAs, (iv) examination of the design space and required control strategies, (v) validation, scale-up, and production [10]. While QbT primarily focused on end-stage testing, QbD revolved around advanced product and process understanding for systematic design of the operating space using mechanistic models and design of experiments (DoE). However, more recently there has been a shift towards quality-by-control (QbC), wherein quantitative and predictive understanding is leveraged for active process control with robust process design and operation, enabling smart manufacturing [11].

A goal of the QbC approach is real-time process monitoring and management, wherein advanced process control strategies are utilized to handle disturbances and exceptional events [11]. Process analytical technology (PAT) methods play a crucial role in monitoring a variety of CQAs in order to accomplish this [8,9]. Monitoring and control of CQAs such as tensile strength and tablet porosity are critical as they are linked to dissolution profiles of the manufactured tablets, which are ultimately linked to patient safety and treatment efficacy [12–16]. Tablet tensile strength and dissolution profile are affected by various factors such as particle size, API concentration, and addition of lubricants and glidant [17,18]. Glidants are added to improve the flowability of the blend. However, glidants and lubricants are also known to impact other product parameters, such as bulk density, compactibility, and compressibility. An objective of this work is to incorporate the impact of the use and control of glidants while assuring that the critical properties, such as tensile strength of the manufactured tablets, are maintained at desirable levels. In the context of continuous manufacturing, when a glidant feeder is used, it is important to use calibrated mechanistic models to handle the variations of glidant concentration. Therefore, it is essential to explore a variety of different control strategies to address the changes in CQAs of a tablet that may arise when glidants are used. It is worth noting that even though these challenges and control strategies are also relevant to lubricants, the development of mechanistic models and relevant control strategies associated with lubricants is beyond the scope of this work.

The identification and handling of plant-model mismatch (PMM) is an important component of any real-time process monitoring and control approach, and it has been an area of interest for decades. PMM can arise in the continuous tablet manufacturing process for a variety of reasons, e.g., the feeder refill step is known to introduce disturbances that affect CMAs such as the bulk density [19–21]. Since this may result in a deviation in the CQAs, PMM needs to be monitored and algorithms to mitigate it need to be developed and implemented. In order to monitor PMM, Harris initially presented a minimum variance-based assessment criterion to assess the condition of the working control loop [22]. This approach has gained popularity, but it is limited to single-input-single-output (SISO) systems [23]. More recently, data-driven methods that examine autocovariance and solve an optimization problem formulated to address the mismatch estimates in MIMO systems have been developed. These methods minimize the discrepancy between the autocovariance of the output and the actual autocovariance of the mean-centered output variable [24–26]. Partial correlation coefficient (PCC)-based methods to identify PMM have also received attention in the literature, where PCC is well-suited as it is able to handle cases with high correlation in the manipulated variables [27,28]. As model re-identification is a critical, and often a time-consuming step once PMM has been identified, hybrid machine learning approaches have been proposed in order to aid model selection [28]. While there is great depth in the literature associated with the identification of PMM, there is limited discussion on practical approaches that would be applicable to the continuous pharmaceutical manufacturing industry in terms of management of the PMM [29,30]. CQAs and CMAs need to be tracked online during plant operation but they may be unmeasurable in practice through existing PAT sensing methods (e.g., the bulk density within a unit operation); therefore, alternative solutions are required. This work proposes novel state estimation methods to accurately track states and model parameters online and, hence, guide operating decisions.

Additionally, most work in the continuous tablet manufacturing domain utilizes linear model predictive control strategies, often resulting from the linearization of a nonlinear system, which may not be adequate for some strongly nonlinear processes [31–33]. A literature review of traditional MPC application for different unit operations in the continuous pharmaceutical manufacturing industry can be found in [34], including end-to-end in silico and implementation studies. Since there is limited implementation of nonlinear model predictive control strategies for the continuous pharmaceutical manufacturing industry, a main objective of this work is to develop and present a moving horizon estimation-based nonlinear model predictive control (MHE-NMPC) framework to serve the dual requirement of accurate estimation and effective control. Model predictive control strategies are also advantageous over traditional proportional-integral-derivative (PID)-based control strategies, as they are able to effectively handle constraints, loop interactions and non-square control systems that may be encountered in manufacturing of pharmaceutical solid dosage forms [35–38]. A main practical concern for any developed framework is the need to ensure that the optimization problem can be solved in real-time, particularly for relatively quick processes such as those in the continuous pharmaceutical manufacturing industry. Therefore, an additional objective is to examine and discuss the real-time feasibility of the developed framework in controlling a rotary tablet press.

It is important to note that once non-conforming quality attributes have been identified, a long-term goal is the integration of control frameworks similar to the MHE-NMPC structure with residence time distribution (RTD)-based modeling frameworks that are currently being developed to guide tablet product diversion in the continuous pharmaceutical manufacturing industry and truly enhance and enable smart manufacturing operations [39,40].

To summarize, the primary objective of this work is to develop and present a moving horizon estimation-based nonlinear model predictive control (MHE-NMPC) framework to serve the dual requirement of accurate estimation and effective control, and to demonstrate its practical applicability by discussing its implementation feasibility in controlling a rotary tablet press. A secondary objective of this work is to examine different control strategies that are required when incorporating glidant feeders to further control tablet properties.

The rest of this work is organized as follows. In Section 2, mathematical modeling and optimization approaches for state estimation and control will be briefly discussed, along with the proposed monitoring algorithm and its advantages, i.e., the MHE-NMPC framework to monitor CMAs and CQAs and determine control actions will be presented. Section 3 will illustrate the robustness of the proposed MHE-NMPC framework with two examples of application. Specifically, the studies will showcase monitoring and control of a rotary tablet press in the presence of (i) plant-model mismatch and (ii) uncertainty in the glidant concentration. Section 4 will provide concluding remarks and directions for future work.

## 2. Material and Methods

### 2.1. State Estimation

State and parameter estimation methods have been utilized to enhance process monitoring capabilities in a number of industrial applications, ranging from bioreactors to robotics to continuous pharmaceutical manufacturing [30,41,42]. State and parameter estimation is a powerful tool in scenarios where process states or model parameters cannot be directly measured with sensors.

A nonlinear state-space model is defined as follows:

(1) $$\dot{x}=g(x,u,\theta ,w)$$

**Figure 1.**
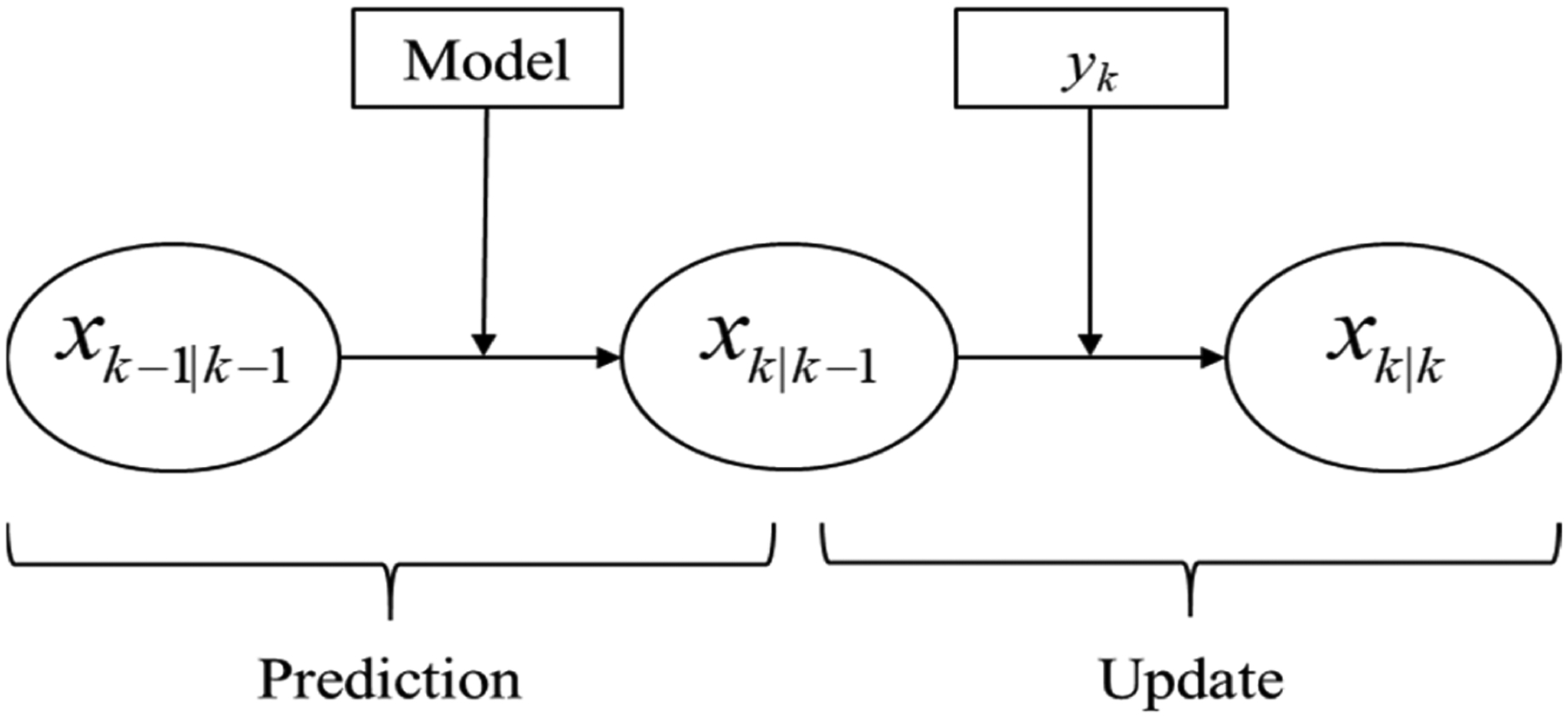
Conventional state estimation algorithm.

(2) y=l(x,u,θ,v)

where *x*, *u*, *θ*, and *y* are the state variable vector, input variable vector, model parameter vector, and measurement vector, respectively [43]. The process and measurement noise are denoted by *w* and *v*, respectively. A schematic illustration of conventional state estimation algorithms is presented in Figure 1, where the nonlinear model is initialized based on the state values at the previous time step (*k* − 1) in order to obtain a prediction of the states and model parameters at the current time (*k*). State measurements are obtained from available sensors and are utilized to obtain a more accurate estimate of the states and parameter values by correcting the predictions from the model.

A number of alternative algorithms to carry out state estimation have been developed, e.g., the Kalman filter (KF), extended Kalman filter (EKF), unscented Kalman filter (UKF), particle filter (PF), and moving horizon estimation (MHE) are among the more popular approaches [44–47]. While the KF and EKF algorithms are suitable for linear or approximately linear applications, the UKF, PF, and MHE algorithms are able to handle processes that are more nonlinear in nature. The KF, EKF, and UKF algorithms also assume that error distributions are Gaussian in nature, while this assumption does not have to be satisfied for the PF and MHE algorithms [46]. Unlike the conventional approach illustrated in Figure 1, MHE utilizes a window, or moving horizon, of previous measurements in order to estimate the current states and model parameters, often providing improved performance when compared to the other algorithms. MHE is also capable of handling measurements collected from sensors at different sampling intervals or frequencies, which is advantageous for industries that utilize a variety of sensors to track physical attributes, as is the case in continuous pharmaceutical manufacturing. Therefore, MHE will be the choice of state estimation algorithm utilized in this work, as it is able to effectively track plant-model mismatch caused by deviations in model parameters, such as variations in the bulk density due to uncertainty in upstream unit operations (e.g., refilling of feeders).

The importance of monitoring powder feeder dosing in continuous pharmaceutical manufacturing is investigated in great detail by Destro and co-workers [29], wherein an MHE-based state estimation approach is implemented to reconcile mass measurements that are available from loss-in-weight (LIW) feeders with downstream measurements that are available from a PAT instrument and, thereby, to obtain practically continuous measurements as opposed to sampled measurements provided from the PAT instrument. The authors also demonstrate that the MHE approach is superior to one that utilizes statistical filters instead of the state estimator. Similarly, robust estimators were incorporated within the MHE skeleton of a feeding-blending system to handle dynamic systems with gross errors [30]. While the results presented in both case studies are promising, neither discusses or elaborates on the importance of their integration with efficient control strategies. To the knowledge of the authors, there has been no work in the continuous pharmaceutical manufacturing domain that has examined the integration of state estimation strategies with efficient control strategies, and therefore, this will be the central focus of this work, as discussed in the following sections.

### 2.2. Model Predictive Control (MPC)—Linear and Nonlinear

Model predictive control methods have been employed by various industries over the past few decades [48–52]. MPC relies on the dynamic model of the process. This model can either be linear or linearized models obtained through system identification, as in the case of the linear implementation of MPC, or be nonlinear and derived using first principles or semi-empirically (using a hybrid model), as in the case of NMPC [53]. Both MPC algorithms utilize a finite time horizon to optimize the control input at the current time iteration, while keeping future time iterations in mind. This ability makes MPC predictive in nature due to its ability to anticipate future events and take control actions accordingly, which is not possible using traditional PID controllers [54].

While nonlinear model predictive control (NMPC) methods have been utilized by some industries, to the knowledge of the authors their implementation has not been explored extensively for continuous pharmaceutical manufacturing of solid dosage forms, cf. [31–33], where linear or hybrid implementations of MPC are utilized. Since processes in the continuous pharmaceutical manufacturing industry are known to be nonlinear in nature, it is therefore desirable to develop and implement an NMPC approach.

It should also be noted that these predictive control strategies are particularly advantageous for cases of non-square systems, i.e., where the number of manipulated variables exceeds the number of the controlled variables, since these methods are able to effectively manage nonlinear relationships [35–37]. These cases cannot be straightforwardly handled using traditional PID control strategies [38].

The following section will present the developed MHE-NMPC framework that seeks to accomplish the dual requirement of accurate estimation and efficient control. Since real-time implementation feasibility is an area of interest, a discussion on the practical applicability of the framework developed will also be presented.

### 2.3. Moving Horizon Estimation-Based Nonlinear Model Predictive Control (MHE-NMPC) Framework

**Figure 2.**
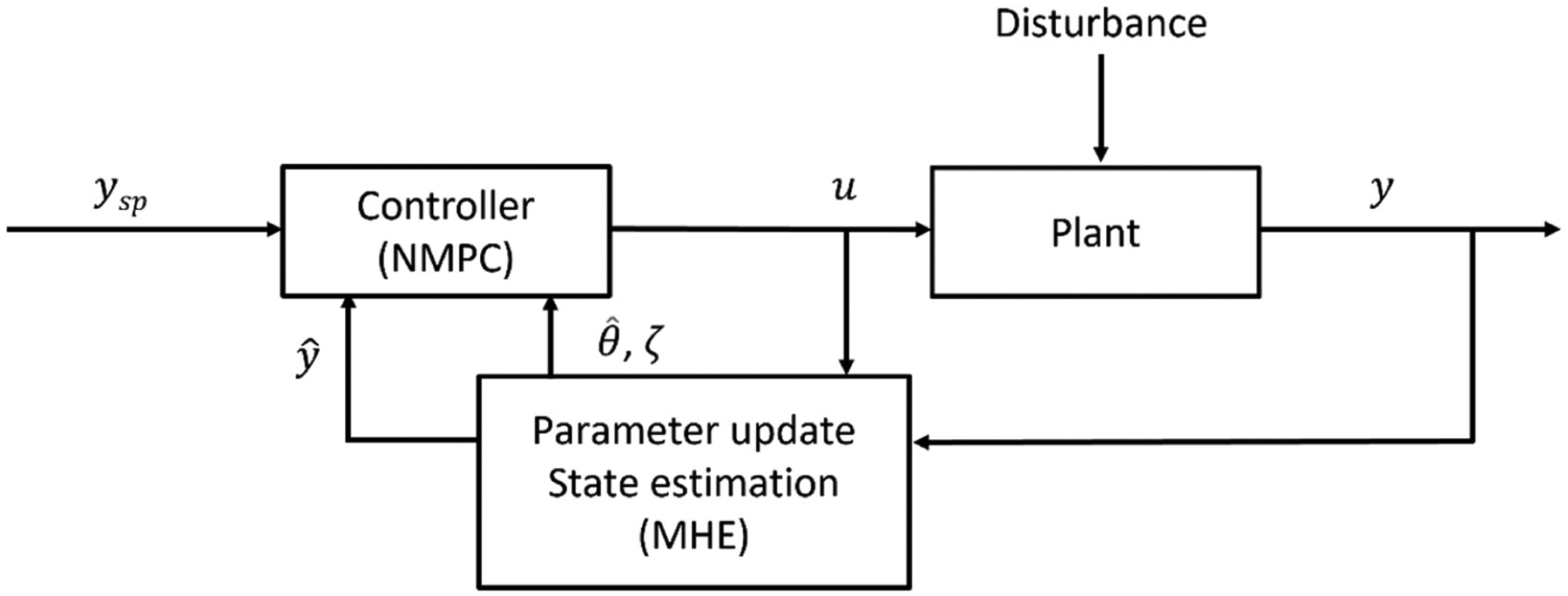
Adaptive control framework of MHE-NMPC.

The algorithm proposed in this work seeks to combine the effective estimation capabilities of MHE with the control abilities of NMPC, through the MHE-NMPC framework illustrated in Figure 2. Real-time measurements of output variables (*y*) and input variables (*u*) are first collected to monitor the process. Since disturbances, either known or unknown, can always exist in a real plant, mismatches may arise between the sensor measurements and model values. As elaborated previously, the goal of state estimation is to obtain a ‘true state’ value by utilizing the information from both measurements and process models. The ‘true state’ can be either measurable, e.g., API concentration at the blender exit using NIR sensors [55–57], or unmeasured, e.g., powder holdup in the blender. Through the updating of uncertain model parameters, which have changes due to upstream disturbances, MHE enables the handling of plant-model mismatch, thus allowing the controller to receive estimated output variables ($\hat{y}$) with less uncertainty. The NMPC control algorithm then minimizes the error between setpoints *y*_*sp*_ and estimated output variables ($\hat{y}$) by deciding the optimal control move (*u*) for the process to reach both setpoint tracking and disturbance rejection, i.e., the control objectives, while updating the model parameter (${\hat{\theta }}_{k}$) and median of the error distribution in the past time window (*ζ*).

**Figure 3.**
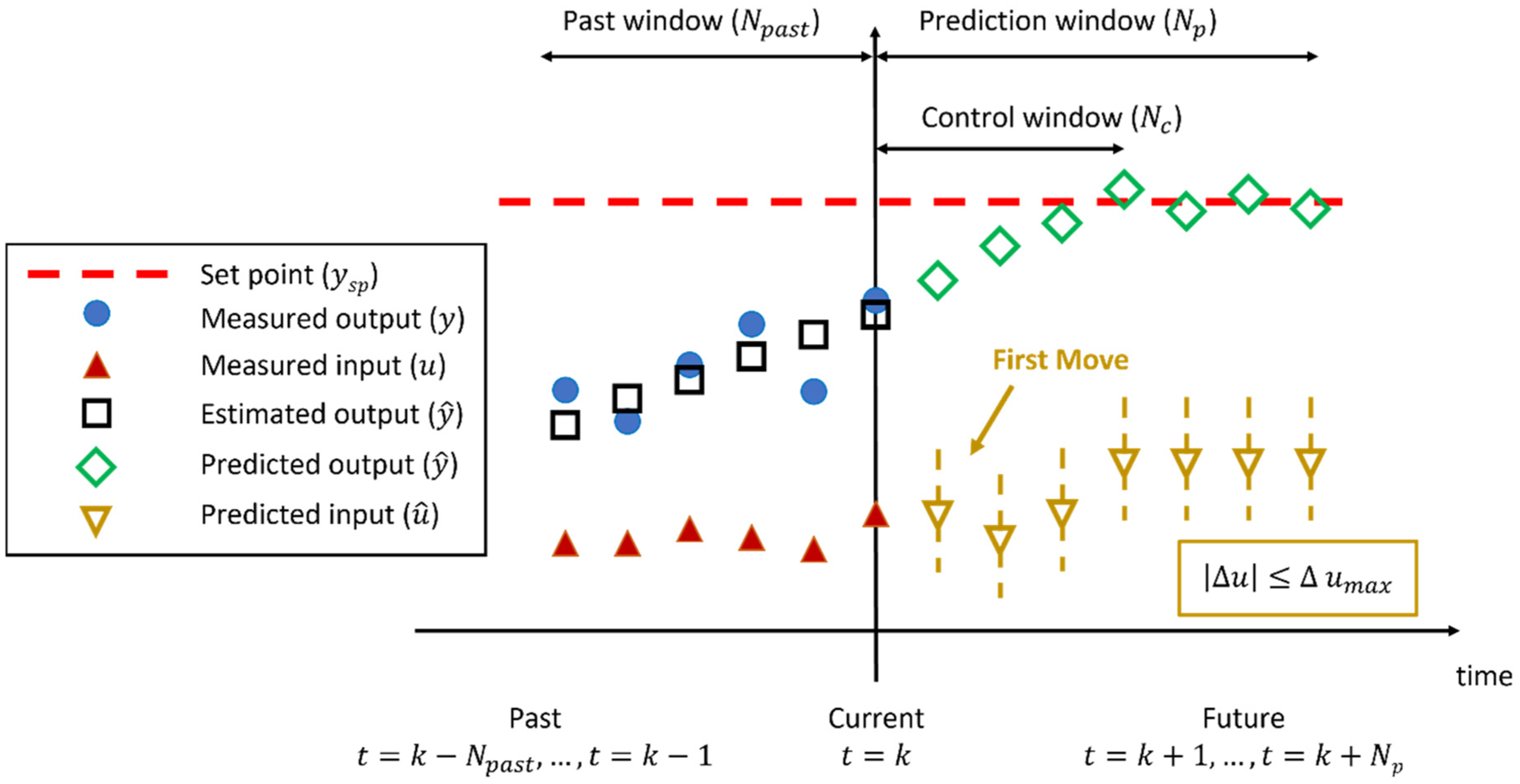
Illustration of MHE-NMPC coupling at each time interval.

A schematic illustration of the MHE-NMPC framework at time *t* = *k* is shown in Figure 3, with *N*_*past*_ measurements available in the past window and *N*_*p*_ estimations in the prediction window. The MHE is then formulated as follows:

(3a) $$\underset{{\hat{\theta }}_{k}}{\text{min}}J=\sum_{t=k-N_{\text{past }}}^{k}{\left(\epsilon_{t}\right)}^{T}W_{E}\epsilon_{t}+{\left({\hat{\theta }}_{k}-{\hat{\theta }}_{k-1}\right)}^{T}W_{\theta }\left({\hat{\theta }}_{k}-{\hat{\theta }}_{k-1}\right)$$

subject to

(3b) $${\hat{x}}_{k-N_{\text{past }}+j+1}=f\left({\hat{x}}_{k-N_{\text{past }}+j},u_{k-N_{\text{past }}+j},{\hat{\theta }}_{k}\right)$$

(3c) $${\hat{y}}_{k-N_{\text{past }}+j}=h\left({\hat{x}}_{k-N_{\text{past }}+j}\right)$$

(3d) $$\epsilon_{k-N_{\text{past}}+j}=y_{k-N_{\text{past }}+j}-{\hat{y}}_{k-N_{\text{past}}}+j$$

(3e) $${\hat{x}}_{k-N_{\text{past }}+j+1}\in X,\;\epsilon_{k-N_{\text{past }}+j}\in \Omega_{\epsilon },\;{\hat{\theta }}_{k}\in \Omega_{\theta }$$

(3f) $$j=0,1,\ldots ,N_{\text{past }}$$

where ${\hat{\theta }}_{k}$ are estimated uncertain parameters, which are bounded in the compact set Ω_*θ*_. In the above formulation, *y*_*t*_ and *u*_*t*_ are measurements of output variables and input variables at time *t*, respectively; ${\hat{y}}_{t}$ and ${\hat{x}}_{t}$ are estimated output and state values, respectively; *ε*_*t*_ are output disturbances, which are bounded in the compact set Ω_*ϵ*_; and *W*_*E*_ and *W*_*θ*_ are the weighting matrices. After the MHE optimization problem is solved at time *t* = *k*, the estimated state ${\hat{x}}_{k}-N_{\text{past }}+1|t=k$ is chosen as the initial state value of next time step *t* = *k* + 1, i.e., ${\hat{x}}_{k-N_{\text{past }}+1|t=k+1}={\hat{x}}_{k}-N_{\text{past }}+1|t=k$ [58].

While an error distribution of output variables $y_{t}-{\hat{y}}_{t}$ in the past time window can be obtained from Equation (3d), a single point estimate of the output ${\hat{y}}_{t}$ is of most interest in many applications, instead of the whole error distribution [59]. When no probabilistic process models are used, it is easier to use a single point estimate of the output ${\hat{y}}_{t}$ to visualize and control process dynamics. In this study, the median of the error distribution in the past time window is used to represent output disturbances *ζ*_*k*_ at time *t* = *k*, i.e.,

(4) $$\zeta_{k}=\text{median}\left\{\epsilon_{k-N_{\text{past }}+j}\right\},\text{ for }j=0,1,\ldots ,N_{\text{past }}$$

Therefore, with estimated states ${\hat{x}}_{k}$, output disturbances *ζ*_*k*_, and updated uncertain optimal parameters ${\hat{\theta }}_{k}$, the NMPC framework at time *t* = *k* is given by:

(5a) $$\underset{\Delta u_{t}}{\text{min}}J=\sum_{t=k}^{k+N_{p}}{\left({\hat{y}}_{t}-y_{sp}\right)}^{T}W_{y}\left({\hat{y}}_{t}-y_{sp}\right)+\sum_{t=k}^{k+N_{c}-1}\left(\Delta u_{t}^{T}W_{\Delta u}\Delta u_{t}\right)$$

(5b) $${\hat{x}}_{k+j+1}=f\left({\hat{x}}_{k+j},{\hat{u}}_{k+j},{\hat{\theta }}_{k}\right)$$

(5c) $${\hat{y}}_{k+j}=h\left({\hat{x}}_{k+j}\right)+\zeta_{k}$$

(5d) $$\Delta u_{k+j}={\hat{u}}_{k+j+1}-{\hat{u}}_{k+j}$$

(5e) $${\hat{x}}_{k+j}\in X,\;{\hat{u}}_{k+j}\in U,\;\Delta u_{k+j}\in \Omega_{\Delta \text{u}}$$

(5f) $$j=0,1,\ldots ,N_{p}-1$$

where *N*_*c*_ is the length of the control time window, and *y*_*sp*_ are the setpoints of the output variables. *W*_*y*_ and *W*_Δ*u*_ are the weighting matrices. Control movements Δ*u* are constrained in the compact set Ω_Δ*u*_. The control window *N*_*c*_ is usually smaller than the prediction window *N*_*p*_ and has to be chosen considering a compromise between computational burden and stability requirements. Control movements Δ*u*_*k*+*j*_ in control window *N*_*c*_ vary according to results of optimization, but those beyond the control window are zero, i.e., $\Delta u_{k+N_{c}}=\Delta u_{k+N_{c}+1}=\cdots =\Delta u_{k+N_{p}-1}=0$, which implies that ${\hat{u}}_{k+N_{c}}={\hat{u}}_{k+N_{c}+1}=\cdots ={\hat{u}}_{k+N_{p}}$. In other words, while the predicted ${\hat{y}}_{k+j}$ can still be calculated using Δ*u*_*k*+*j*_ and ${\hat{u}}_{k+j}$ in the prediction window *N*_*p*_, only Δ*u*_*k*+*j*_ in control window *N*_*c*_ is considered in the objective function. It should be noted that models of estimated output variables ${\hat{y}}_{t}$ are different in Equations (3c) and (5c). In the future time window, output disturbances *ζ*_*k*_ are added to the model of the process, allowing zero steady-state offset in controlled output variables *y* [59,60]. A schematic illustration of MHE-NMPC is provided in Figure 3, where at each iteration, the MHE is utilized to obtain a more accurate representation of the true state of the process and plant-model mismatch, and the NMPC is utilized to find the optimal first move for each input variable *u*. This framework thus allows for both accurate estimation and efficient control.

### 2.4. Implementation of a Real-Time Feasible MHE-NMPC Framework

The MHE-NMPC framework is implemented in MATLAB (MathWorks R2018a) and the MATLAB built-in *fmincon* function is used to solve the optimization problem in each iteration. The computation is performed on a 64-bit ASUS VivoBook with Intel^®^ Core^™^ i7–8550U @1.80 GHz processor and 8GB of total memory. In all simulated results, the time unit for each step is 1 s, the past time window *N*_*past*_ used in MHE is 30 time units, and the NMPC is tuned with prediction time window *N*_*p*_ chosen to be 60 time units and control time window (*N*_*c*_) to be 10 time units. Sensor measurements are also assumed to be available at 1 s intervals. It should be noted that the average computation time for each iteration is 0.7 s, indicating that the optimization problem can be solved and implemented in real time. These results demonstrate the feasibility of the proposed framework, and its ability to achieve real-time process control.

The following section will explore the applicability of the developed MHE-NMPC framework to track plant-model mismatch and to efficiently control a key process unit operation in the continuous manufacturing line of solid dosage forms, i.e., the rotary tablet press.

## 3. Examples of Application to Continuous Direct Compression

The applicability of the developed MHE-NMPC framework will be demonstrated through two case studies. The first case study will highlight the importance of monitoring model parameters in real time and how this is enabled via state estimation, as opposed to the use of fixed model parameters. Different degrees of plant-model mismatch will be used. The second case study will present the applicability of the framework in the practical scenario of having uncertainty in the glidant feeding conditions.

**Figure 4.**
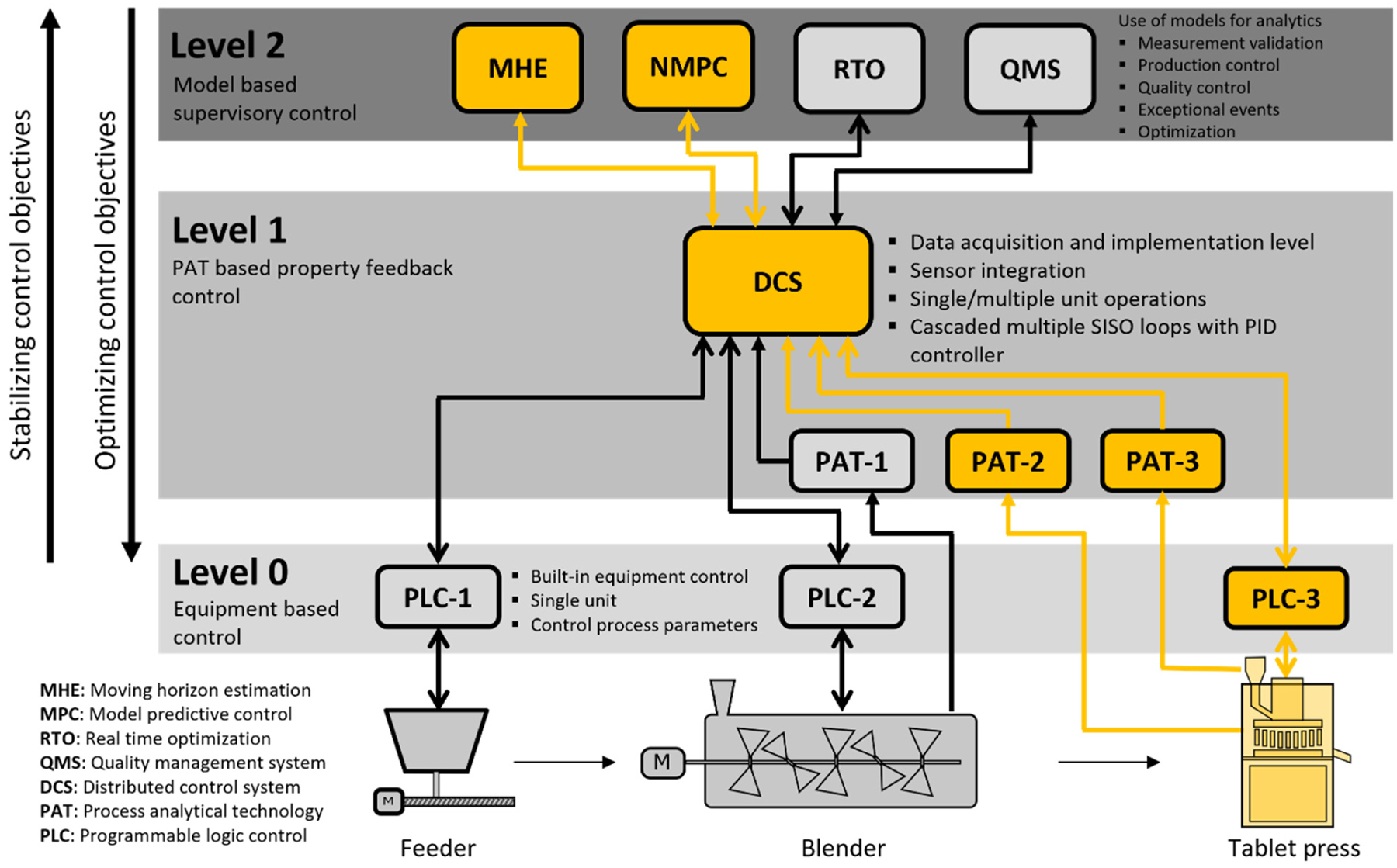
A 3-level hierarchical implementation of control systems for the continuous direct compression process (modified from source: [11]).

Both case studies will focus primarily on the tablet press unit operation of the direct compression line. A hierarchical implementation of the three-level quality-by-control (QbC) framework of control systems for the continuous direct compression line is illustrated in Figure 4, whose unit operations are comprised of feeders, blenders, and the tablet press. For this line, Level 0 control is generally implemented through programmable logic control (PLC) systems built into the unit operation equipment in order to control CPPs. Level 1 control relies on PAT tools to monitor and control CQAs and can encompass multiple unit operations designed to reduce the impact of disturbances that may propagate downstream. Level 1 control supervises the Level 0 control, typically accomplished through SISO control loops which aim to maintain desired setpoints for the CQAs. A distributed control system (DCS) is employed to integrate process equipment such as the feeders and tablet press and any instrumentation that measures material properties. More advanced approaches are applied at Level 2 and use mathematical models such as MHE for validating process measurements, with the ability to predict the effects of disturbances and changes in the CPPs on the CQAs. Additional functionalities provided at Level 2 can include NMPC, a quality management system (QMS), and real-time optimization (RTO).

### 3.1. Tablet Press Model

The rotary tablet press and the lubricant/glidant feeder are key unit operations, where the latter is used to reduce frictional loses and facilitate powder flow during die filling and formation of solid tablets via mechanical compression. Therefore, models for glidant effects in die filling and compression processes will be used to monitor and control the porosity and tensile strength of tablets. Specifically, these mechanistic models capture the effects of glidant concentration and mixing conditions [61,62].

The weight of a convex tablet, *W*, formed using Natoli D-type tooling with shallow cup depth can be computed as follows:

(6) $$W=\rho_{b}V_{\text{fill}}\left(1-\xi_{1}\frac{n_{T}}{n_{F}}+\xi_{2}\frac{H_{\text{fill}}}{D}\right)$$

where *D*, *V*_*fill*_, *H*_*fill*_, *ρ*_*b*_, *n*_*T*_, and *n*_*F*_, refer to the diameter of the die, volume of the die cavity, dosing position, bulk density of the powder, turret speed, and feed frame speed, respectively [62]. In Equation (6), *ξ*_1_ and *ξ*_2_ are model parameters to be estimated from experimental data. The bulk density depends on glidant concentration and mixing conditions, but its dependency is beyond the scope of this work. For the D-type tooling, the volume of the die cavity is given by:

(7) $$V_{\text{fill}}=\frac{\pi D^{2}H_{\text{fill}}}{4}+\frac{\pi h\left(\frac{3D^{2}}{4}+h^{2}\right)}{6}$$

where *h* is the cup depth. The tablet production rate, *m*_*tablet*_, is given by:

(8) $${\dot{m}}_{\text{tablet }}=Wn_{T}N_{\text{station }}$$

where *N*_*station*_ is the number of turret stations available. For a blend composed of MCC (Avicel PH200), APAP (acetaminophen) and silica experimental evidence suggests that both pre-compression and main compression forces do not show a dependency on glidant conditions [62]. The pre-compression force (PCF) is then computed as follows [63]:

(9) $$F_{pc}=\frac{\pi D^{2}}{4b}\left[\frac{\rho^{pc}-\rho_{c}}{\rho^{pc}(a-1)+\rho_{c}}\right]$$

where parameters *a* and *b* are Kawakita constants [63], which represent the maximum degree of compression and the reciprocal of the pressure applied to attain this degree of compression, respectively. In Equation (9), *ρ*_*c*_ and _*ρ*_^*pc*^ refer to the critical density and the pre-compression relative density, respectively. The pre-compression relative density is computed as follows:

(10) $$\rho^{pc}=\frac{W}{V^{pc}\rho_{t}}$$

and

(11) $$V^{pc}=\frac{\pi D^{2}H^{pc}}{4}+\frac{\pi h\left(\frac{3D^{2}}{4}+h^{2}\right)}{3}$$

where _*ρt*_ and *H*^*pc*^ refer to the true density of the powder and the pre-compression thickness, respectively. Similarly, the main compression force (*F*_*punch*_) is computed as follows:

(12) $$F_{\text{punch }}=\frac{\pi D^{2}}{4b}\left[\frac{\rho^{\text{in}-\text{die }}-\rho_{c}}{\rho^{\text{in}-\text{die }}(a-1)+\rho_{c}}\right]$$

with the in-die relative density _*ρ*_^*in*−*die*^ given by:

(13) $$\rho^{\text{in}-\text{die}}=\frac{W}{V^{\text{in}-\text{die}}\rho_{t}}$$

and

(14) $$V^{in-die}=\frac{\pi D^{2}H^{in-die}}{4}+\frac{\pi h\left(\frac{3D^{2}}{4}+h^{2}\right)}{3}$$

where *H*^*in*−*die*^ refers to the main compression thickness. The tablet density, or out-of-die relative density of the tablet, _*ρ*_^*tablet*^, is then obtained utilizing the elastic recovery, *ε_ρ_*, of the tablet as follows:

(15) $$\rho^{\text{tablet }}=\left(1-\varepsilon_{\rho }\right)\rho^{\text{in}-\text{die }}$$

The elastic recovery model is not sensitive to the glidant mixing conditions [61,62], and it is governed by:

(16) $$\varepsilon_{\rho }=\varepsilon_{0}\frac{\rho^{\text{in}-\text{die}}-\rho_{c,\varepsilon }}{1-\rho_{c,\varepsilon }}$$

where *ε*_0_ and *ρ*_*c*,*ε*_ are the in-die elastic recovery at full compaction and the relative density at which tablets do not exhibit elastic recovery, respectively [64]. The tensile strength *σ*_*t*_ exhibits dependency on glidant conditions and it is computed as follows

(17) $$\sigma_{t}=\sigma_{0}\left[1-\left(\frac{1-\rho^{\text{tablet }}}{1-\rho_{c,\sigma_{t}}}\right)e^{\left(\rho^{\text{tablet }}-\rho_{c,\sigma_{t}}\right)}\right]$$

where *σ*_0_ is the tensile strength at zero porosity and $\rho_{c,\sigma_{t}}$ is the critical relative density at which tablets do not exhibit any the tensile strength, i.e., the relative density at which a tablet starts forming [17]. It bears emphasis that these parameters are functions of glidant conditions, specifically:

(18) $$\rho_{c,\sigma_{t}}=\frac{\rho_{c,0}-\rho_{c,\infty }}{1+C_{\sigma }}+\rho_{c,\infty }$$

(19) $$\sigma_{0}=\frac{\sigma_{0,\phi }}{1+C_{\sigma }}$$

(20) $$C_{\sigma }=\frac{c_{l}^{b_{1}}\gamma^{b_{2}}}{b_{3}}$$

where *ρ*_*c*,0_, *ρ*_*c*,∞_, *σ*_0,*ϕ*_, *b*_1_, *b*_2_, and *b*_3_ are model parameters estimated from experimental data. In Equation (20), *c*_*l*_ and *γ* are the glidant concentration and total shear imparted to the blend, respectively. For simplicity, the total shear strain is represented by an equivalent mixing time, which, in turn, is estimated as follows

(21) $$\gamma =\gamma_{0}+\frac{m_{f,h}}{{\dot{m}}_{\text{tablet }}}$$

where *γ*_0_ and *m*_*f*,*h*_ are a total shear strain base line, expressed in term of mixing time, and the mass hold up in the feed-frame and hopper, respectively. Specifically, for the purpose of these case studies, the model parameter *γ*_0_ is the glidant mixing time used in the rotary Tote blender when the blend was prepared. A 5L rotary Tote blender was employed. The mean residence time in the feed-frame, i.e., $\frac{m_{f,h}}{{\dot{m}}_{\text{tablet }}}$, is used to estimate the additional shear, or mixing time, imparted inside the tablet press.

The dependency of the bulk density on the glidant concentration, *c*_*l*_, can be incorporated through the following equation [62]:

(22) $$\rho_{b}=\rho_{b,\infty }-\frac{\rho_{b,\infty }-\rho_{b,0}}{1+C_{\rho }}$$

where *ρ*_*b*,∞_ and *ρ*_*b*,0_ represent the bulk densities when the shear strain imparted is infinite and zero, respectively. *C*_*ρ*_ is a lumped parameter that defines the glidant mixing conditions computed as follows [62]:

(23) $$C_{\rho }=\frac{c_{l}^{r_{1}}{\left(\gamma +\gamma_{0}\right)}^{r_{2}}}{r_{3}}$$

where *γ* and *γ*_0_ are the shear imparted to the powder during mixing and the initial shear strain imparted prior to mixing, respectively. *r*_1_, *r*_2_, and *r*_3_ are fitting parameters.

A Natoli NP-400 tablet press and SOTAX AT4 tablet tester were used in this work to fabricate tablets and gather experimental data under steady-state conditions. The experimental data were then used to carry out parameter fitting using the *fmincon* function in MATLAB to obtain realistic model parameters values that could be used for the simulations presented in case studies 1 and 2, which are summarized in Table 1.

### 3.2. Case Study 1: Monitoring and Control of the Rotary Tablet Press in the Presence of Plant-Model Mismatch

Monitoring powder bulk density in the tablet press is of critical importance, as it affects the tablet properties [12]. Sources of variability can be introduced during any of the unit operations upstream, e.g., in the feeder unit operations during refill, as the feeder switches from gravimetric mode to volumetric mode, leading to either increases in bulk density due to compression or decreases in bulk density due to aeration [19–21].

For this case study, a four-by-five system was employed as it would enable the incorporation of an extra manipulated input for added control benefits, i.e., glidant concentration. It is assumed that the direct compression line has the ability to utilize the glidant concentration as a manipulated variable through changes in the glidant flowrate. In practice this would be implemented in the hierarchical three-level QbC framework, by using a level-one PID control, that would use the glidant concentration measurement and adjust the glidant flowrate to follow the concentration setpoint set by the level-two NMPC. In this case the four-by-five non-square level-e control system is comprised of controlled variables consisting of the tablet weight, pre-compression force, production rate, and tensile strength and manipulated variables consisting of the dosing position, pre-compression thickness, main compression thickness, turret speed, and glidant concentration. It is assumed that measurements for the tablet weight, pre-compression force, main compression force, and production rate are all available every second [61]. In this simulation, it should be noted that the main compression force was not a directly controlled variable with specified set points. This is because the tensile strength could not be maintained while simultaneously fixing the main compression force. Given the objective to maintain the tensile strength at desired levels due to its link to patient safety, the tensile strength was chosen over the main compression force as a controlled variable. Since measurements of the main compression force are available, they were utilized in the MHE framework only for the purpose of parameter estimation. As maintaining the CQAs is important, and since the tensile strength measurements are not available in real time, a soft sensor based on Equation (17) is utilized in order to track this particular state in real time. In practice, the SOTAX AT4 tablet tester can be utilized in order to obtain measurements of the tensile strength. However, since the diametrical compression test is destructive, tensile strength measurements are available at a lower frequency than one of the PAT sensors, which in turn also drives the need for a soft sensor.

A summary of the controlled variables, manipulated variables, measured variables, and uncertain model parameter is provided in Table 2. In order to examine the performance of the MHE-NMPC framework under PMM, three different scenarios will be examined, namely: nominal operation (no PMM), operation with mild PMM, and operation with high PMM. A summary of model parameters for each scenario is provided in Table 1, where the MHE-NMPC tuning parameters are those described in detail in Section 2.4. Mild PMM is simulated by introducing mismatch to three model parameters: *ρ*_*b*_, *ρ*_*c*_ and Kawakita parameter *a*. High PMM is simulated by introducing mismatch to six model parameters: *ξ*_2_, *ρ*_*b*_, *ρ*_*c*_, Kawakita parameters *a* and *b*, and *ρ*_*t*_. In this simulation, the ‘model’ and ‘plant’ share the same equations detailed in Section 3.1. Different parameter values were assigned to the ‘model’ and ‘plant’ in order to simulate mismatch. Additionally, sensor measurement noise in the plant was simulated by adding normally distributed error with zero mean and variance analogous to the variability of a real sensor. This variability was obtained from historical plant data.

For all three scenarios, three model parameters are tracked, i.e., *ρ*_*b*_, *ρ*_*c*_ and Kawakita parameter *a*. The bulk density was monitored due to its influence on a number of other model parameters and states, while the relative critical density and Kawakita parameter *a* are both known to influence the compression forces, making them critical parameters that also need to be tracked in real time.

**Figure 5.**
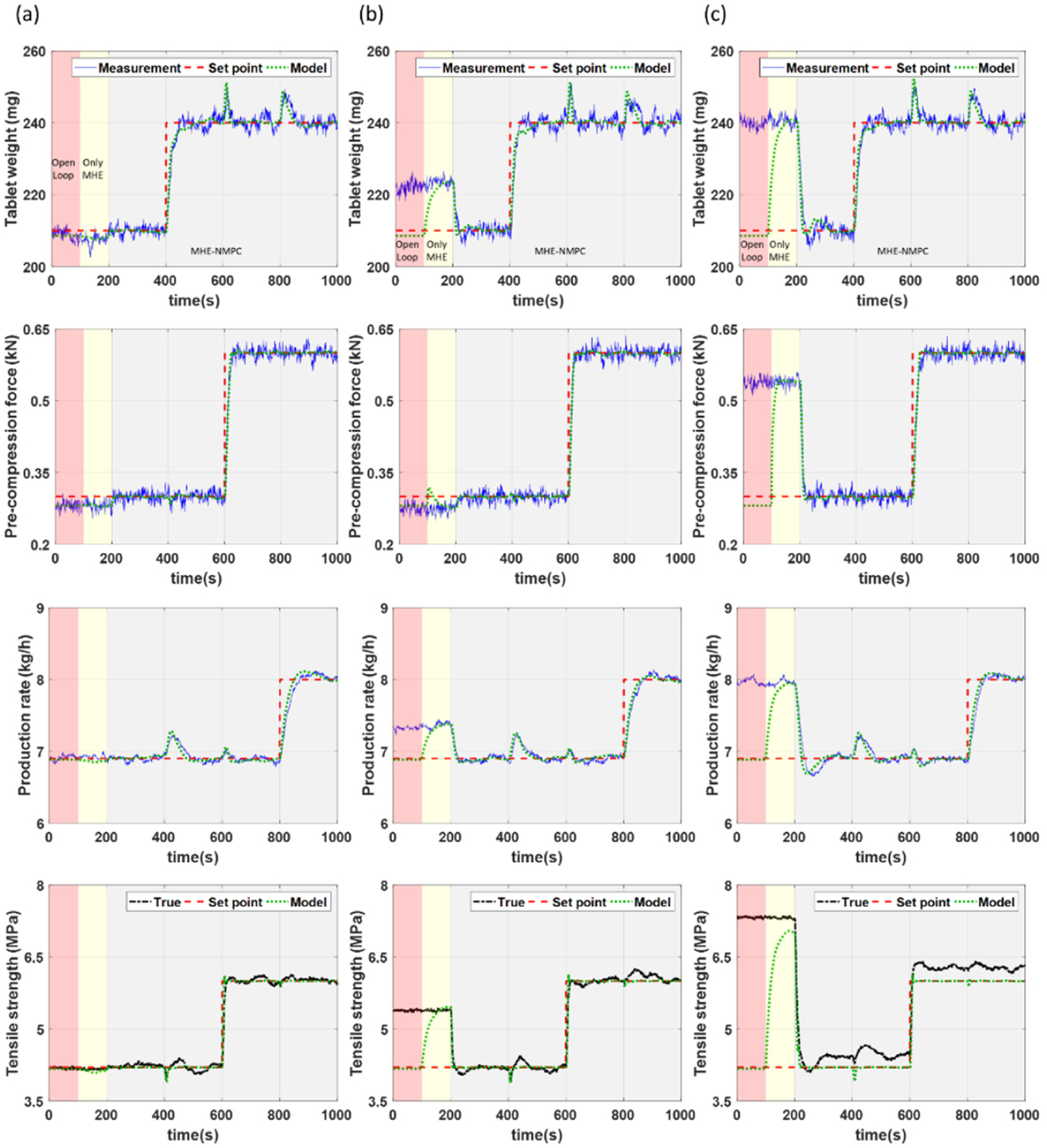
Controlled variables for case study 1 under scenarios with (**a**) no PMM, (**b**) mild PMM, and (**c**) high PMM.

Simulation results of the process outputs for all three scenarios are presented in Figure 5a–c, respectively. The MHE-NMPC framework is utilized for all simulations and includes open-loop control from 0–100 s (indicated by red shading in all plots), state estimation using MHE and open loop control from 100–200 s (indicated by yellow shading in all plots), and implementation of the MHE-NMPC framework from 200 s until the end of the simulation (indicated by gray shading in all plots). Setpoint changes are introduced for the tablet weight from 210 mg to 240 mg at 400 s, for the pre-compression force from 0.3 kN to 0.6 kN at 600 s, for the production rate from 6.9 kg/h to 8 kg/h at 800 s, and for the tensile strength from 4.2 MPa to 6 MPa at 600 s, respectively.

**Figure 6.**
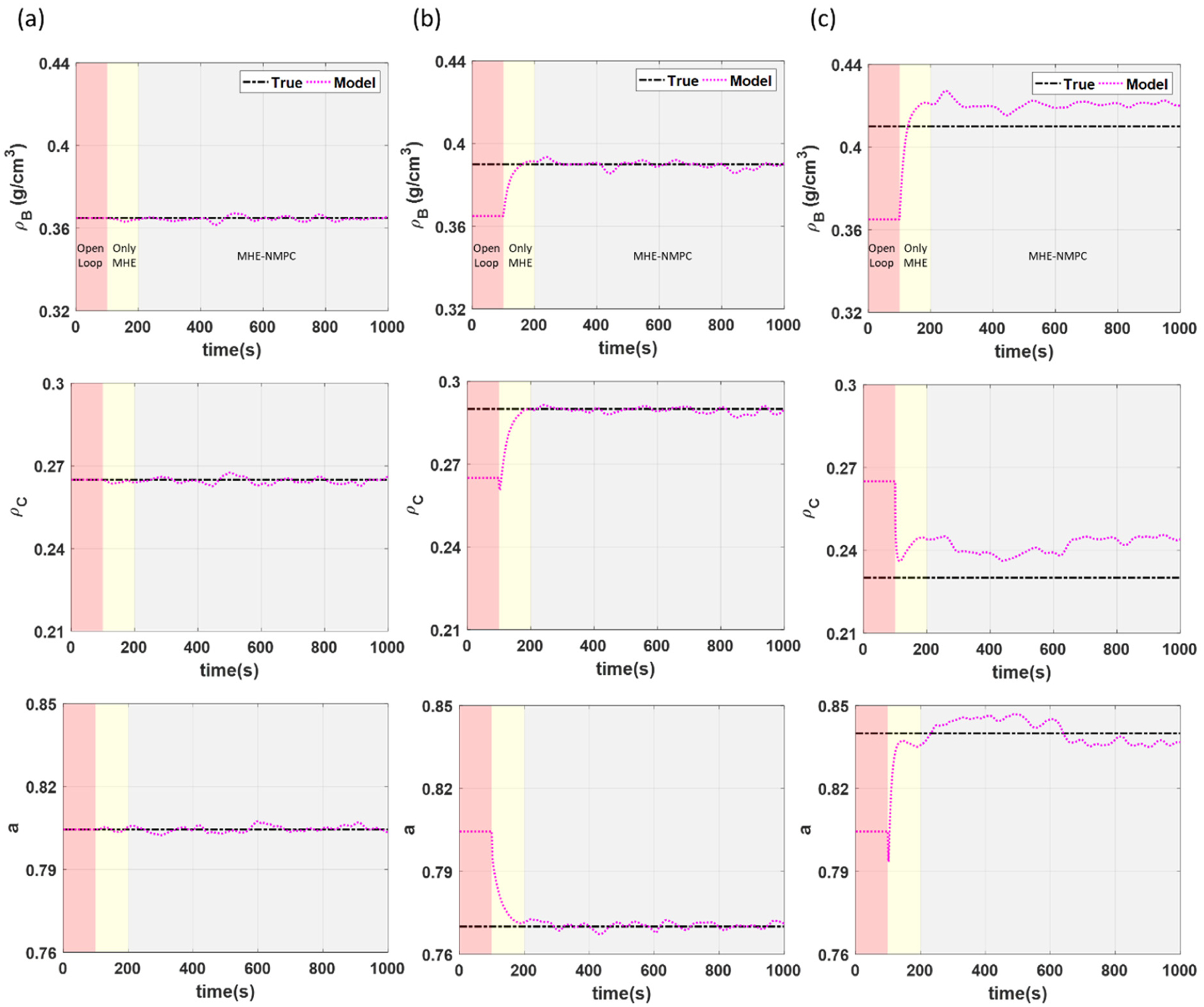
Parameter estimation of model parameters for case study 1 under scenario with (**a**) no PMM, (**b**) mild PMM, and (**c**) high PMM.

**Figure 7.**
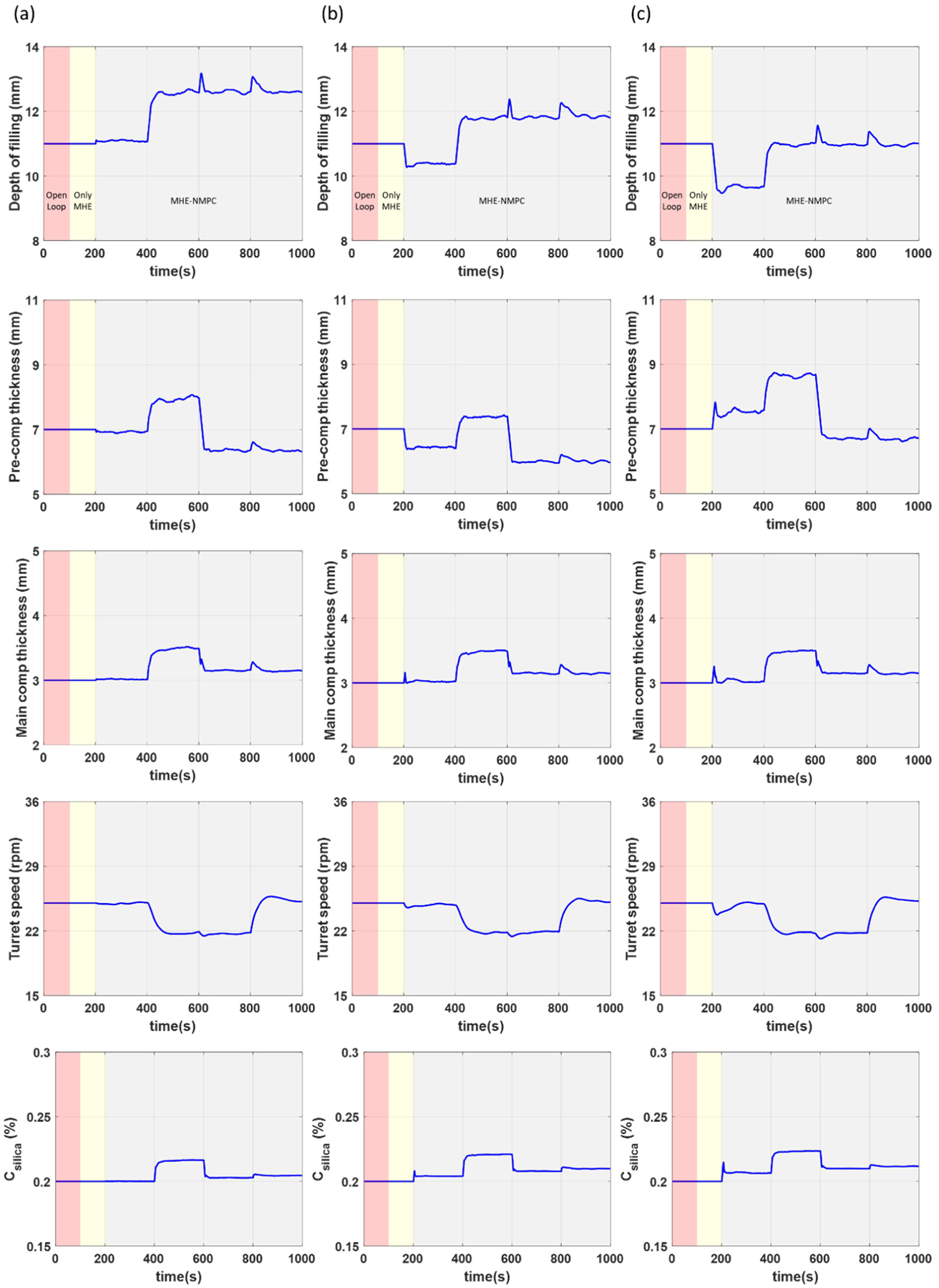
Manipulated variables for case study 1 under scenarios with (**a**) no PMM, (**b**) mild PMM, and (**c**) high PMM.

For the scenario where there is no PMM, accurate setpoint tracking can be achieved for all states (see Figure 5a). This is also true for the case where there is mild PMM (see Figure 5b). This is primarily enabled due to the ability of the MHE-NMPC framework to accurately track variations in the uncertain model parameters in real time as illustrated in Figure 6b, allowing the impact of PMM to be effectively managed. In the case where there is high PMM (see Figure 5c), fairly accurate setpoint tracking can still be achieved despite there being mismatch in more parameters than those being tracked. This is also observed from the parameter estimation results in Figure 6c, although there is a slight offset in the model parameters being tracked to compensate for the variation in the additional model parameters that are not being tracked. The corresponding plots of the manipulated variables for all three scenarios are presented in Figure 7.

Control performance metrics are required in order to assess the performance of the development framework and accurately assess the impact of PMM for each scenario. Beyond the typical controller performance metric, i.e., integral of absolute error (IAE), some additional metrics are used to quantify the control performance in each scenario, which include the duration-to-reject (D2R) and magnitude to product (M2P) [65]. These metrics are able to quantify control performance in a manner that is more relevant for the continuous pharmaceutical industry. D2R is the duration of time that the process requires to smooth out the process disturbance or to reach a new set point for the CQA/CPP. M2P describes the maximum deviation in the CQA/CPP from the target setpoint. Larger values of all these performance metrics indicate worse or degraded control performance. The IAE values are calculated from t = 300 s to t = 1000 s. A summary of the control performance metrics is provided in Table 3.

When mild PMM exists, the control performance of the MHE-NMPC framework is comparable to the scenario without PMM, implying that the framework is able to sufficiently handle the PMM. However, when there is excessive PMM in multiple parameters as in the scenario with high PMM, significantly higher values of IAE and M2P are obtained, particularly in the tensile strength. This can be attributed to the fact that there was mismatch in more model parameters than those being tracked for this particular simulation, as can be noted from Table 1, resulting in an offset in the estimates of the model parameters to compensate for the added uncertainty (see Figure 6c). It should also be noted that the setpoints for the tablet weight and production rate track reasonably well, even in the presence of high PMM as demonstrated by the comparable IAE values for both states. However, since the tensile strength is an important CQA that is linked to the dissolution profile of the tablets, once high PMM begins to cause an offset in the tensile strength, it can serve as an indicator for the requirement to carry out model re-identification. This case study was able to demonstrate the strength of the MHE-NMPC framework in its ability to handle PMM in multiple model parameters.

### 3.3. Case Study 2: Monitoring and Control of the Rotary Tablet Press in the Presence of Uncertainty in the Glidant Concentration

This scenario aims to explore a more practical concern with regards to the incorporation of the glidant feeder in the control scheme. In practice in some applications, it might not be possible to accurately control the concentration of the glidant in the direct compression process, due to the low concentrations used and, thus, the small feeding rates needed. Uncertainty in the glidant concentration is important, as it leads to variations in the bulk density upstream of the rotary tablet press. Therefore, accurate monitoring and control of these variations is required.

Since the glidant concentration can no longer be treated as a manipulated input for this scenario, the original system is revised to form a four-by-four MIMO system with the controlled variables consisting of the tablet weight, pre-compression force, production rate, and tensile strength. The manipulated variables consist of the dosing position, pre-compression thickness, main compression thickness, and turret speed. Once again it is assumed that only measurements for the tablet weight, pre-compression force, main compression force, and production rate are available every second. As tensile strength measurements are not available in real time, a soft sensor based on Equation (17) is once again utilized for this particular state. The concentration of silica is then assumed to be an uncertain parameter. A summary of the controlled variables, manipulated variables, measured variables, and uncertain model parameters is provided in Table 4. A summary of the model parameters was provided in Table 1, where the MHE-NMPC tuning parameters are those detailed in Section 2.4.

**Figure 8.**
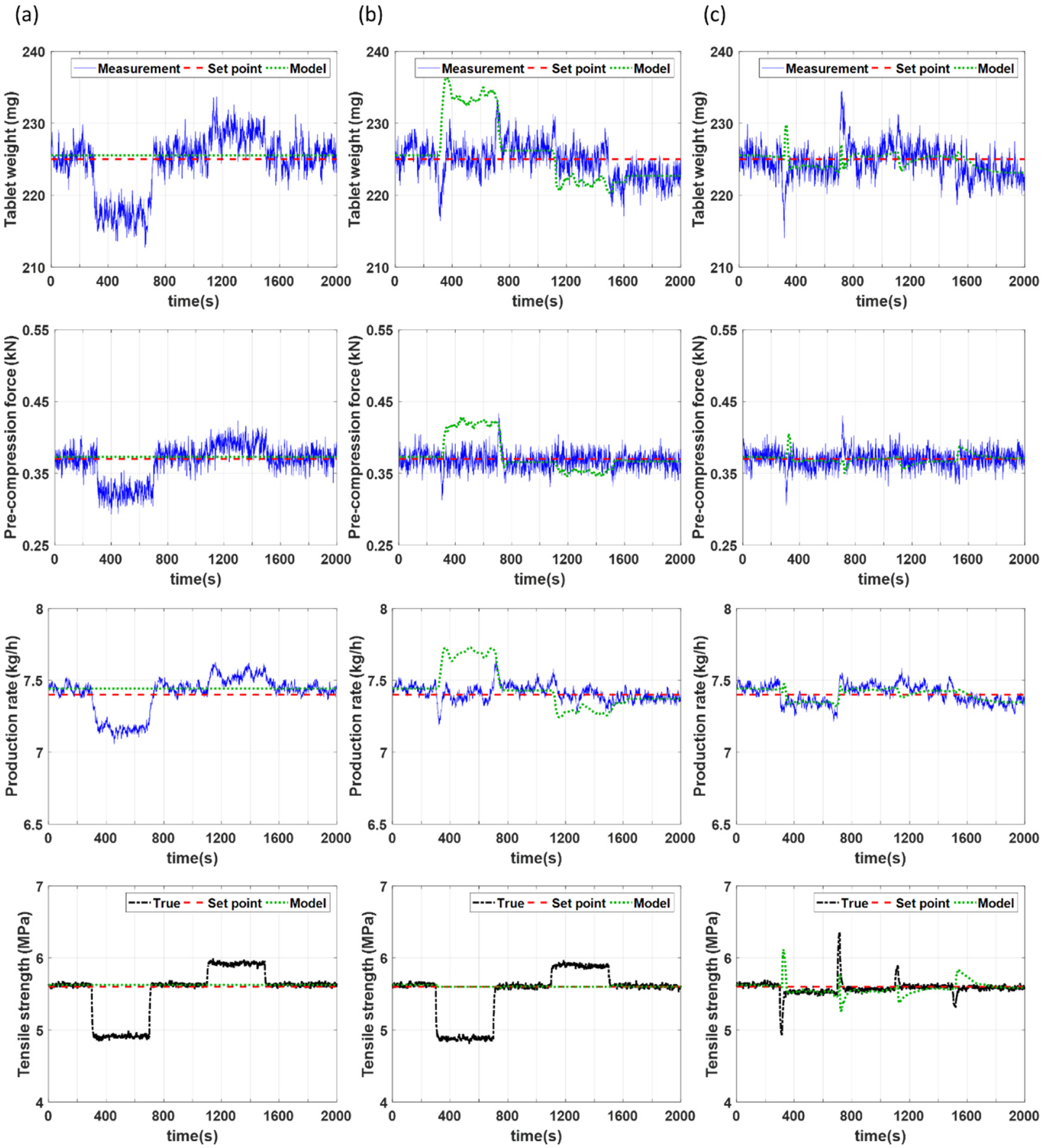
Controlled variables for case study 2 under scenarios using (**a**) open loop control, (**b**) only NMPC, and (**c**) MHE-NMPC.

For this particular case study, mismatch is introduced through positive and negative step changes in the silica concentration from its nominal value of 0.2% to 0.35% between 300 and 700 s and from 0.2% to 0.05% between 1100 and 1500 s, respectively. Step changes in either direction are introduced in order to examine if and how the direction of the mismatch affects the control performance. Simulation results of the process outputs for open loop control, closed loop control using only NMPC control, and closed loop estimation and control using the proposed MHE-NMPC framework are presented in Figure 8a–c, respectively. The simulation for the NMPC framework includes open loop control from 0 to 200 s, with a closed loop NMPC strategy implemented from 200 s until the end of the simulation. The simulation for the MHE-NMPC framework includes open loop control from 0 to 100 s, state estimation using MHE and open loop control from 100 to 200 s, and implementation of the MHE-NMPC framework from 200 s until the end of the simulation.

Figure 8a demonstrates failure of the open loop control to maintain the controlled variables at their setpoints and, thus, the need to implement effective control strategies. Due to the disturbance terms utilized in the NMPC framework, offset free control is achieved for three of the four controlled variables when only NMPC is employed, as illustrated in Figure 8b. However, since real-time measurements are unavailable for the tensile strength, a soft sensor is employed. Since the soft sensor does not incorporate a disturbance term, an offset can be observed between the soft sensor values and the set point due to the mismatch caused by the assumption of fixed model parameters in the NMPC framework. In contrast, as illustrated in Figure 8c the MHE-NMPC framework is able to achieve offset free control for all four states, including the tensile strength. This is attributed to the ability of the MHE-NMPC framework to provide (i) real-time and accurate estimation of uncertain model parameters, enabled by MHE, and (ii) efficient control, enabled by NMPC.

**Figure 9.**
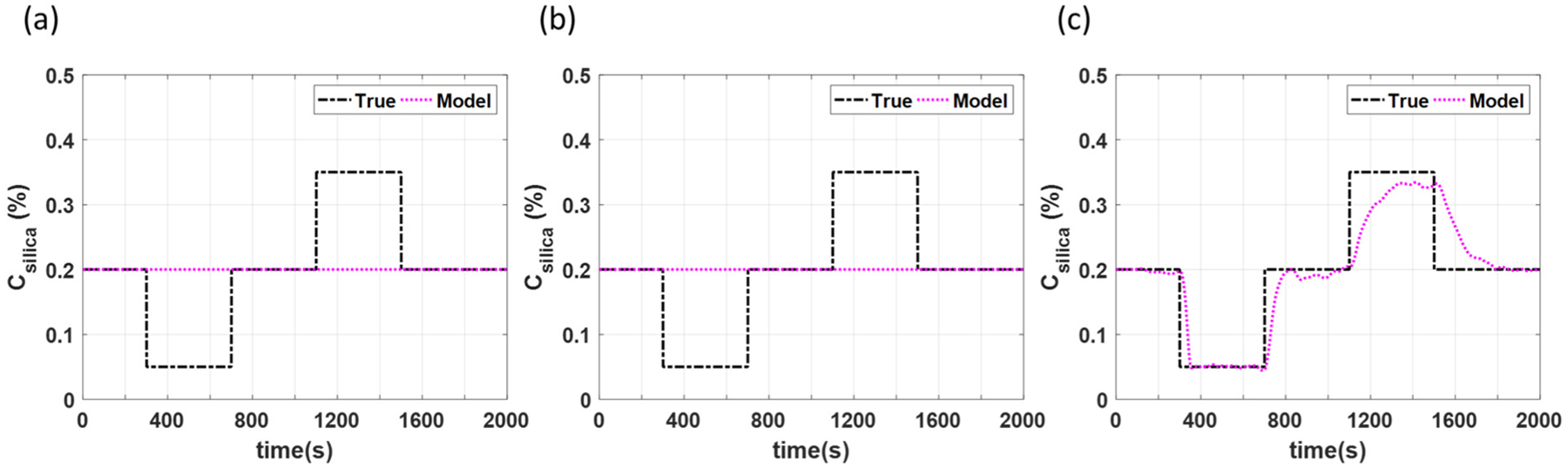
Parameter estimation for case study 2 under scenarios using (**a**) open loop control, (**b**) only NMPC, and (**c**) MHE-NMPC.

Figure 9 shows results for the uncertain model parameter estimation, i.e., the estimation of silica concentration. These results demonstrate the ability of the MHE-NMPC framework to accurately track variations in silica concentration. It should be noted that the sluggish behavior at higher concentrations of silica is due to the nonlinear effect silica has on the process. Since in practice the true value of the concentration of silica might be unknown, this case study demonstrates the advantage of utilizing the MHE-NMPC framework to achieve accurate estimation of both measurable and unmeasurable states and parameters, such as the concentration of silica, while executing realistic and effective control strategies.

**Figure 10.**
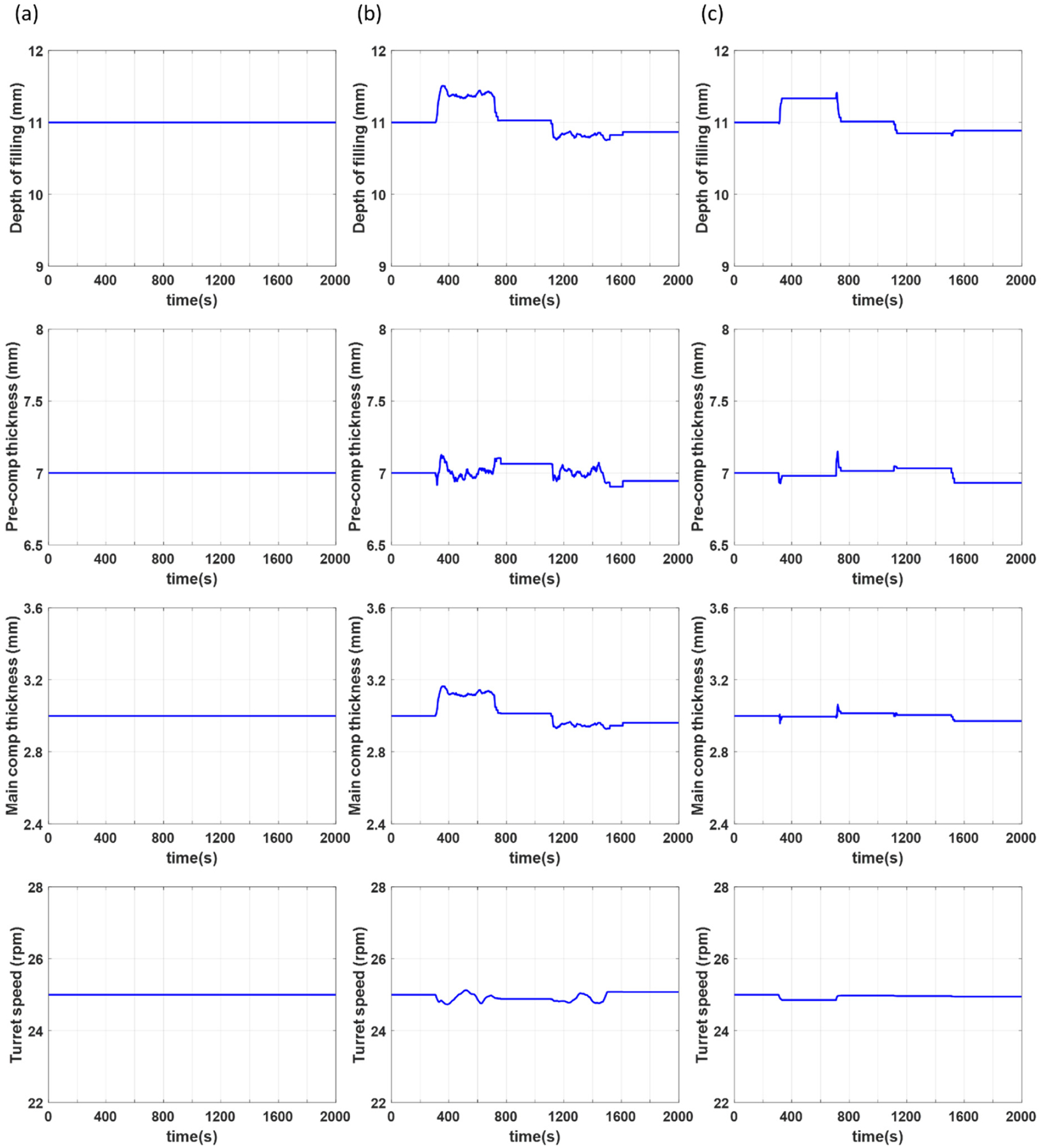
Manipulated variables for case study 2 under scenarios using (**a**) open loop control, (**b**) only NMPC, and (**c**) MHE-NMPC.

Figure 10 shows the manipulated variables for the different control strategies studied in this section. All changes in the manipulated variables presented in these results are realistic in nature and can be achieved during normal operation of the tablet press. It should be noted that the variations in the manipulated variables are larger in the case where only NMPC is utilized (see Figure 10b) compared to the case where the MHE-NMPC framework is utilized (see Figure 10c). This may be attributed to a less effective linear output disturbance model implemented in the NMPC framework, when compared to the MHE-NMPC framework that also carries out parameter updating incorporating more directly nonlinear effects of the PMM in the scheme.

This case study was able to demonstrate the ability of the MHE-NMPC framework to track and manage fluctuations in the glidant concentration, often caused by upstream disturbances, thereby providing an efficient solution to a common process upset faced when operating the rotary tablet press.

## 4. Conclusions

The continuous pharmaceutical manufacturing industry is in need of improved real-time process monitoring and management strategies that, specifically, are able to effectively identify and handle plant-model mismatch (PMM). In order to enable the quality-by-control (QbC) paradigm to move forward, this work developed and presented a moving horizon estimation-based nonlinear model predictive control (MHE-NMPC) framework to accomplish the dual requirement of accurate estimation and efficient control. Real-time implementation feasibility of the developed framework was also discussed, and the ability of the proposed framework to solve the optimization problem at each time step in a manner that enabled real-time implementation was highlighted. The practical applicability of the developed framework was corroborated through two realistic case studies that incorporated the effects of glidant to better control CQAs such as the tensile strength. Both examples demonstrated the ability of the framework to achieve reasonable control performance despite the presence of varying sources and degrees of PMM.

Future work includes further demonstration of the practical applicability of the proposed MHE-NMPC framework utilizing the rotary tablet press at Purdue University, including the application of the framework to the entire direct compression line. While a soft sensor was utilized in this work to track the tensile strength, in practice, due to low-frequency measurement availability from the SOTAX AT4 tablet tester, sensor fusion methods might be required to integrate and efficiently utilize all available plant data. This improved strategy would also require additional studies to determine how frequently to collect measurement data from the SOTAX AT4 tablet tester, due to the destructive nature of the testing method.

## Figures and Tables

**Table 1.** Summary of model parameters for case studies 1 and 2.

	Case Study 1			Case Study 2
Purpose	Assess Control Performance in the Presence of Different Levels of PMM			Assess Control Performance When Uncertainty in Glidant Concentration Is Present
Assumption	Glidant Concentration Can Be Manipulated			Glidant Concentration Needs to Be Estimated
Model Parameters	No PMM	Mild PMM	High PMM	Nominal Operation
ξ_1_	0.036	0.036	0.036	0.036
ξ_2_	0.030	0.030	0.050	0.030
*ρ*_*b*_ (g/cm^3^)	0.365	0.390	0.410	0.365
*ρ*_*c*_	0.265	0.290	0.230	0.265
Kawakita: *a*	0.80	0.77	0.84	0.80
Kawakita: 1/b (MPa)	10.26	10.26	8.55	10.26
*ρ*_*t*_ (g/cm^3^)	1.53	1.53	1.51	1.53
*ε*_0_	0.08	0.08	0.08	0.08
*ρ*_*C*,*ε*_	0.57	0.57	0.57	0.57
*σ*_0_ (MPa)	11.67	11.67	11.67	11.67
*ρ*_0_	0.57	0.57	0.57	0.57
*ρ*_∞_	0.61	0.61	0.61	0.61
*b*_1_	0.31	0.31	0.31	0.31
*b*_2_	0.38	0.38	0.38	0.38
*b*_3_	8.40	8.40	8.40	8.40
*ρ*_*b*,∞_, (g/cm^3^)	N/A			0.450
*ρ*_*b*,0_ (g/cm^3^)				0.330
*r*_1_				0.361
*r*_2_				1.394
*r*_3_				23.326

**Table 2.** Summary of variables and uncertain model parameters for case study 1.

**Controlled variables**	Tablet weight, pre-compression force, production rate, tensile strength
**Manipulated variables**	Dosing position, pre-compression thickness, main compression thickness, turret speed, silica concentration
**Measured variables**	Tablet weight, pre-compression force, main compression force, production rate
**Uncertain model parameters**	Bulk density, critical density, *a*: maximum degree of compression

**Table 3.** Control performance of the MHE-NMPC framework for case study 1 under different levels of PMM.

Controlled Variables	Performance Metrics	No PMM	Mild PMM	High PMM
**Tablet Weight**	IAE	6.83	7.00	7.05
	M2P (%)	3.31	3.19	3.61
	D2R (s)	76	78	74
**Tensile Strength**	IAE	9.95	10.18	39.07
	M2P (%)	5.25	5.23	10.46
	D2R (s)	82	81	90
**Production Rate**	IAE	8.84	8.26	8.41

**Table 4.** Summary of variables and uncertain model parameters for case study 2.

**Controlled variables**	Tablet weight, pre-compression force, production rate, tensile strength
**Manipulated variables**	Dosing position, pre-compression thickness, main compression thickness, turret speed
**Measured variables**	Tablet weight, pre-compression force, main compression force, production rate
**Uncertain model parameters**	Silica concentration
